# Supplementary material for: Continuous or discrete magnitudes? A comparative study between cats, dogs and humans
Source: PLoS One. 2025 Oct 1;20(10):e0331924. doi: 10.1371/journal.pone.0331924 (PMC12488003; doi:10.1371/journal.pone.0331924)
Supplement: S1 File — (DOCX) [file pone.0331924.s001.docx]

**Supplementary Information**

**Continuous or discrete magnitudes? A comparative study between cats, dogs and humans**

Mireia Solé Pi^1¶^, Luz A. Espino^1¶^, Péter Szenczi^2,3^, Marcos Rosetti^1,3*^, Oxána Bánszegi^1*^,

**S1. Instructions for human participants**

After checking that the participants met all inclusion criteria, the experimenter gave them the next instructions verbally before starting the test (originally in Spanish):

“In this test we want to evaluate the perception of different images. The test will be performed on a computer, where you will be presented with two images and you will have to choose one of them according to the instructions given. The whole process should take about 5 minutes. It’s important that you know that your participation in this study is voluntary and that you can decide to stop at any moment. If you have any questions regarding the points mentioned or require additional details, feel free to tell me and I will offer you help.

Once the participants had signed the consent form and they were seated in front of the computer, the PsychoPy test began, which showed the next instructions on screen:

“We will present two images, one on your right side and one on your left side. You must choose the image that has the LARGER black area. Try to answer as fast as you can.”

After completing each trial, there was a transitional white screen with an orange dot in the center that the participants had to press, with the purpose of recalibrating the mouse to the center of the computer screen. The following instruction was shown each time this screen appeared: “To see the next image, click on the ORANGE circle.”
