# Supplementary material for: Continuous or discrete magnitudes? A comparative study between cats, dogs and humans
Source: PLoS One. 2025 Oct 1;20(10):e0331924. doi: 10.1371/journal.pone.0331924 (PMC12488003; doi:10.1371/journal.pone.0331924)
Supplement: Attachment — Submitted filename: Review.pdf [file pone.0331924.s003.pdf]

Great job improving the clarity of the manuscript! Overall, the authors did a careful revision and adequately responded to my previous comments. The procedure is much clearer. However, I still have some comments and suggestions but much minor compared to the previous ones. As a general comment, the authors tend to use sentences and paragraphs that are too big and with too many ideas (this is even more salient in the discussion).

Abstract

p-2, line 31-32: be more specific than “some preference” or, at least, specify a preference for what.
p.2, line 34: at this point, the reader does not know what is the control condition. Instead of using the name of the condition, describe it as you did in the rest of the sentence.

Introduction

p. 4, line 68: processing numerical information involves a larger cognitive load compared to what? Also, what “this strategy” is referring to? You did not describe any strategy in the previous sentence.
p.4, lines 73 to 76: Divide the sentence in key ideas. As it is, it is difficult to understand the message.
p.4, line 82: why do you considered the use of number rather than of the continuous magnitudes a bias?
p.5, line 91: species rely on the ratio between what? the numerosity? the surface area? of the two stimuli. The information inside brackets is important to understand the results so it should be a regular sentence.
p.5, line 97: decompose the sentence, there are too many ideas and information. How did the animals choose (response) and what was the correct response? Choosing the three item-set? End the sentence at this point. Then you can start the next sentence with results. This is only a suggestion:” Cats reliably learned to choose (...). But, when surface area was controlled, rats were indifferent between the two stimuli suggesting their previous preference was based on the surface are and not on quantity.”
p.5, line 100: consider substituting “above 0.5 ratios” for “when the ratio between the two stimuli was greater than 0.5”. In the same sentence, I did not understood what the authors meant with “both numerical and continuous magnitude differences”.

p.5, line 105: Explain what you mean by “counterbalanced” in this case.

p.6, line 114: The clarity of this paragraph can be increased.

Methods

p.6, line 127 to 129: weight at least 10 kg instead of “over 10kg” and the information that comes next should not be part of the same paragraph. Suggestion: “This last constraint serve to….”.

p. 7, line 141: “details of”

Table 1: I believe there is a better way to present the table. Since there is much more information regarding cats and dogs, I would suggest a table for those and the information regarding the humans can either be in a separate, simpler table or in the text. Below there is a table suggestion with only some information just to exemplify. You may need to switch the lines with columns due to the space occupied when more info is added.

	Age		Sex			
	M (SD)	Range	Female		Male	
			Neutered	Intact	Neutered	Intact
Cats	6.4 (3.7)	1 – 14	9	1	12	4
Dogs	4.1 (3.1)	1 – 13	11	4	9	4

p. 10, line 189: the ratio between the surface areas of the two food portions was 0.5

p.14, line 249: the experimenter asked the participant’s initials, age….

p. 14, line 260: what is S1?; “completed trials of three different conditions”

p.14, line 263: “a total of 28 trials, **x** of each condition”

p.15: Four trials of Control A plus 4 of Control B plus 6 of Experimental do not comprise 28 trials as stated before. I anticipate there were two trial-blocks?!

p.15, line 272: Suggestion: “If participants took more than 10 s to click on either option (i.e., the maximum choice latency allowed), the trial was cancelled and the next trial started immediately.

Results

Instead of “latency of choice”, use “latency to choose”. Be careful when using “female dogs’ choice for the larger” or “human participants’ choice of the larger”, it adds complexity to the information.

Discussion

In general, paragraphs are too long and with too many ideas. Try to divide them.

p.23, line 426: substitute extremely by significantly
p.23, line 428: According to the first part of the sentence, I was expecting the second part to be something like: while cats did not showed a preference for any particular shape or while cats were indifferent between shapes.
P23, line 430: “in the control condition” can be eliminated because you already said it in the beginning of the sentence.
p.25, line 463: did you meant within each sample?.
p.25, line 466: where the **ratio** between…
p.25, line 466: The difference in preference observed between male and female dogs…
p.25, line 467: Did you check if there was any effect similar to that reported by Simona, Maria?
p.25, line 473: dividing **by sex**; it might **be** possible; I think the conclusion of the sentence is odd. I would rather say that any differences may not be possible to observe in your sample because of its small size, mainly when dividing by sex.
p.25, line 476: the discrimination is not between ratios is between two stimuli with a certain ratio between them. Be careful with this among the entire manuscript.
p.25, line 479: please explain what is the result suggests each of the two possibilities. What led you to those possibilities? It seems you are jumping a step in the rationale.
p.26, line 490: …and their **ratio** change which….
p.26, lines 496 and 497: three repetitions of the word making.
p.26, line 498: I believe words are missing from the sentence.
p.27, line 504: latencies
p.27, line 506 and 507: when the control trials **involved** circles of different areas
