# Supplementary material for: Continuous or discrete magnitudes? A comparative study between cats, dogs and humans
Source: PLoS One. 2025 Oct 1;20(10):e0331924. doi: 10.1371/journal.pone.0331924 (PMC12488003; doi:10.1371/journal.pone.0331924)
Supplement: Attachment — Submitted filename: Response to Reviewers.docx [file pone.0331924.s004.docx]

5. Review Comments to the Author

Please use the space provided to explain your answers to the questions above. You may also include additional comments for the author, including concerns about dual publication, research ethics, or publication ethics. (Please upload your review as an attachment if it exceeds 20,000 characters)

Reviewer #1: The manuscript presents a set of experiments using somewhat analogue procedures across three different species (cats, dogs and humans) and aimed to test whether subject’s choices in a spontaneous choice paradigm were mainly controlled by numerosity or surface area of the stimuli. Each subject was tested with three different trial types that the authors named Control Condition A – the two stimuli had the same shape but one had a larger amount of food than the other (ratio = 0.5) –, Control Condition B – similar to A but the ratio was equal to 0.67 – and Test Condition – the two stimuli had the same food amount but arranged in different shapes (four different possible arrangements). I think the research question and the experiments are interesting and I congratulate the authors for their attempt to compare different species. First, I will provide some general comments and then, more specific comments and suggestions.

General Comments:
- The introduction and discussion sections appear to diverge on their focus. The introduction gives a great emphasis on theories and studies addressing does theories thus, leading the reader to expect that the aim of the study was to contrast the theories. I suggest to focus the introduction on previous studies with cats, dogs and humans that try to address the question of control by different stimuli properties – for instance, it would be appropriate to describe the studies you refer in the methods’ section. Moreover, one advantage of this approach is that it would prepare the reader to for your procedure.

ANSWER: We have restructured the introduction and put more emphasis on previous studies (lines 64-73).

- To improve clarity, it might be helpful to reorganize the methods’ section. Nevertheless, I believe that if you address the first point, it will also help.

ANSWER: We reorganized the section as THE REVIEWER suggested below. PLEASE SEE methods Section.

- Figure 2 illustrates the procedure in a really nice way. I was thinking that it may be helpful to make it appear earlier in the manuscript.

ANSWER: Done. LINES 170.

- In the same line, I think the results section would be easier to follow if you divide it by species and not by conditions. In case you maintain it by conditions, use the same names you used before.

ANSWER: we would like to keep the description of the result section in this way because we think it is easier for the readers to follow and compare the performance of the species in the different conditions.

- Consider identifying the test you are using when describing the results. Something like “Binominal tests were conducted to compare… and showed that…”.

ANSWER: WE HAVE ADDED THE NAME OF THE TESTS WHEN DESCRIBING THE RESULTS, WE HOPE THIS IMPROVES THE CLARITY (LINES 334, 337, 339, 343, 346, 349, 352, 356).

- Discussion and Conclusion do not need to be separate sections. You can name it, for instance as “Conclusion and Discussion” or just “Discussion” and end it with a conclusion.

ANSWER: We have removed the “Conclusion” header and merged both sections.

- Be consistent in terminology. For example, use either numerosity or numerousness.

ANSWER: Thank you for your suggestion. We have chosen to use numerosity consistently.

- Be careful when using “test”, “testing session” and “testing procedure” to describe an entire experimental session/ procedure. Because you used the name test for one of the conditions, at the first, it seems that each condition took place in a different session and testing sessions correspond only to the sessions where animals were exposed to the test condition.

ANSWER: We have modified the terms to clarify their meanings and use distinct terms to refer to each of the categories mentioned by the reviewer.

- Do not use the word “random” to describe choice. It is more informative to say that choice was at chance level or did not differ from chance. Notice that animals might be indifferent between the two options because for them, their value is the same. This result would be similar to chance but is not it is not random.

ANSWER: The reviewer is correct. We have replaced instances of random accordingly (LINES 335, 337, 344, 350 and 352)

- The clarity of the data analysis could be enhanced by rephrasing it.

ANSWER: THE DATA ANALYSIS HAS BEEN REPHRASED, WE HOPE THIS IMPROVES ITS CLARITY (LINES 290-325).

Specific Comments:
Abstract
- The first sentence is too general. You can say something like: “A long-standing question in the study of quantity discrimination is what stimulus properties are controlling choice. While some species….”.

ANSWER: Thank you for the suggestion. We have modified the text accordingly (LINE 26).

- The sentence “dogs did not differentiate between either of these ratios” does not make much sense to me. I believe you want to state that dogs did not differentiate between the two options (smaller vs. larger amount of food) regardless of the ratio between them.

ANSWER: Correct. We have amended the sentence (LINES 33-34).

- Keywords: add the species, maybe spontaneous choice; Remove those that are not the focus of the study (e.g., last resort hypothesis).

ANSWER: Thanks for the suggestion. We have added the keywords provided (lines 44-45).

Introduction
- If the focus remains on the theories (see general comment), they should be better explained providing references for each of them and behavioral studies that test them should be described.

ANSWER: WE NOW PROVIDE A LONGER DESCRIPTION OF THE BEHAVIORAL STUDIES SUPPORTING THE THEORIES, AND THE CORRESPONDING REFERENCES (LINES 64-73, 94-102)

- Provide specific examples in cases as this (p.3, line 56): “certain magnitudes (…), others…”.

ANSWER: Good suggestion. We have added an example (LINE 57-58).

- p. 3, line 74: substitute “later on” for “from now on”

ANSWER: Done (LINE 65).

- p.4, line 84: studies test animals’ preferences through choice and not animals’ choice.

ANSWER: Done (LINE 85).

- p. 5, first paragraph: Maintain the order of the species across sentences: first cats and then, dogs (as in the first sentence).

ANSWER: Corrected.

- p.5, line 97: What you described as Weber’s law is only part of it and it is usually known as the distance effect – the greater the distance between two stimuli, the easier it is to discriminate between them (e.g., it is easier to discriminate between 2 and 6 than between 2 and 3). The Weber’s law combines this distance effect with a size effect and states that discrimination is ratio-dependent: pairs of numerical stimuli with the same ratio are equally easy to discriminate. As an example, discriminability is equal between 2 and 6 and between 3 and 9 because the two pairs have the same ratio (r = 3). Moreover, the greater the ratio the easier it is to discriminate between a pair. That is, it is easier to discriminate between 3 and 12 (r = 4) than between the pairs in the previous example.

ANSWER: We have opted to remove the mention of Weber’s law and keep the point of using the ratio to discriminate in order to keep the sentence clear (LINES 98 AND 99)

- p. 5, line 102: Go slower and give more details on studies that are central to your own study.

ANSWER: We have added some detail to the mentioned studies and an additional reference to support the background of the current work (LINES 94-102).

- p.5, line 113: use “vs.” instead of “or”

ANSWER: The use of “vs” is not appropriate in this case, but we have amended the sentence (LINE 120).

- p.6, line 117: use “choice” instead of “decision”; substitute “we included humans” by something like “we also conducted an experiment with humans”.

ANSWER: Done (LINEs 121 and 124)

Method
- Regarding the structure of the Methods’ section (see general comment above), I found it difficult to understand the experimental setup without knowing what was the task of the animals/humans. I am not completely convinced that my suggestion is the better option (see below), it is possible that you may find a better one.
o Subjects (it is more appropriate to use subjects when referring to non-humans).
♣ Cats and Dogs
♣ Humans
o Procedure (join the description of the stimuli within the description of the task and how data collection took place).
♣ Cats and Dogs
♣ Humans
o Data Analysis

ANSWER: We reorganized the methods section as SUGGESTED by the Reviewer. We also direct the reader's attention to the figures at the beginning to the description which might help to understand the experimental setup.

- I suggest the use of numerical digits, in particular when accompanied by a unit (e.g., kg, hr, s).

ANSWER: Great suggestion. We have amended it accordingly THROUGHT THE MS.

- p.6, line 134 – 136: I would recommend to be more specific: What behaviors were observed in each species that signaled that the animals were “nervous/anxious”? How did you measure a lack of motivation (e.g., not approaching food?)? Also, substitute “occasions of testing” by trials.

ANSWER: We have provided examples of signals showing the animals nervous/anxious state but think that the lack of motivation is clear stated. We also replaced the term as suggested (lines 145-147)

- p. 7, line 138 – 140: Make sure that the criteria used is well described. Suggestion: “Data from 3 cats and 6 dogs was not included in the data analysis because the animals showed a lateral bias. That is, they choose the option presented in one side (left or right) in at least five out of y trials on three consecutive testing sessions or in at least 11 out of 14 trials in one session.”

ANSWER: We have added a sentence similar to the reviewer’s suggestion (lines 148-150)

- p. 7, lines 141 to 148: this information can be presented in a table.

ANSWER: We have added a table to present the demographic information for the subjects. SEE TABLE 1, LINE 152.

- p. 7, line 152: how did you recruited the human participants? Email? At classes?

ANSWER: Done. Line 158.

- p. 7, line 154: change “be between 18 and 26 years old” to “have between…”

ANSWER: This and other small corrections were done in these lines (159-160)

- p. 7, line 155: You should add information regarding how did you assessed if the inclusion criteria were met (online questionnaire? Or other?).

ANSWER: Done. Lines 161.

- In my opinion, the excluded participants should go at the beginning of the results’ section. In particular, when excluded after data collection.

ANSWER: Since the excluded participants were not included in the data analysis and the results’ section REFERS TO the final data SET, we would like to keep our description as it is now. HOWEVER, we added an extra sentence to the beginning of the result section to summarize the number of animals which were visited, tested and included in the final analysis (See lines 332-333).

- p. 7, line 159: As it is, the mean age seems to refer only to the women participants. Consider also referring the minimum and maximum age.

ANSWER: this information is now on table 1.

- p. 8, line 166: I think there is probably a mistake in the first two sentences because they are in disagreement. The authors started by saying that they consider the type of reinforcement important. But the next sentence says that previous studies found the type of food had no effect on performance. Taken the two sentences together, the following sentence (line 168) does not make sense.

ANSWER: The reviewer is correct, and we have amended the sentence (lines 176-180).

- p. 8, line 169: Describe in greater detail the procedure to test what was the preferred food. Was it a three-choice? Did you equal the amount? Were the three plates in the same order for all cats?

ANSWER: We added detail to this sentence in lines 177-182.

- p.8, line 171: Data collection involved 28 and not 25 cats since the other 3 were only excluded due to a lateral bias during data analysis.

ANSWER: We corrected this number. It was a typo (line 181)

- p.8, lines 177 – 180: This paragraph is confusing. Are tasks and conditions the same thing? Maybe this information would be easier to understand if it appeared after explaining the task. And, at that point, explain each condition separately and what was its purpose. We can also say something like “Each animal completed one experimental 14-trial session that comprised three different trial types: control A, control B and test trials.” Then, proceed explaining details on each trial type.

ANSWER: As mentioned above we modified the terms to clarify their meanings and use distinct names for each of it. We also reorganized the methods section and direct the reader’s attention to the figures at the beginning of the description of the procedures.

- p.9, line 184: Refer to the Figure before starting its description and use the letters in the text (i.e., A instead of 1, B instead of 3, etc…). Make sure each stimulus is named the same in the text and in the caption of the figure.

ANSWER: Great suggestion. Done. See lines 190-192.

- p. 10, line 216: For replication purposes, I think it is important to make the exact instructions given to human participants available (for instance, as an appendix or even in the manuscript).

ANSWER: We have included the instructions given to the participants in a SUPPLEMENTARY MATERIAL (reference in line 272-273).

- p. 12, line 259: I suggest saying “…to avoid the use of environmental cues.” Also, when using example (e.g.,), there is no need for the use of “etc…” at the end.

ANSWER: Done. Line 256.

- p. 13: Figure 2 shows the experimental setup and not the testing setup (again, it suggests you are referring to the test condition). The details in the caption of the figure should go on the text.

ANSWER: We have changed testing for experimental and summarized the caption to avoid repeating information that was already in-text. See figure 2.

Results
- While the text refers to percentages, you use proportions in the graphs. I suggest you use the same in both, either percentages or proportions.

ANSWER: Done. See lines 334 and 343.

- For the binomial tests, it might be useful to report the 95% Confidence Interval.

ANSWER: Thank you for the suggestion, we have added the confidence interval for each binomial test. Lines 336-337, 338, 345-346, 352 and 425.

- In case of a significant Wilcoxon test, you should report the effect size. It is more conventional to use W instead of V for the test value.

ANSWER: We have reported the effect size for the significant Wilcoxon tests and adopted the W naming convention. Lines 348, 399, 400 and 411.

- Use only two decimals for p-values. If p is less than .001, just report it as p < .001.

ANSWER: We replaced were appropriate (line 336), left as is where the value was equal to .001.

- p. 334, line 318: in this task there is no correct choice. Thus, you should refer to percentage of choosing the larger (see also, line 328).

ANSWER: Done. Lines 340-341.

- p. 16, line 325: Why did you report the humans’ results as proportion of smaller choices? It is confusing given that you started the sentence saying you compared choice of the larger shape to chance levels, that you reported preference for larger for cats and dogs and, that the figure uses proportion of choice of the larger stimuli.

ANSWER: We corrected the proportions to reflect the choice of the larger stimulus. Lines 350-357.

- Figure 3: First, the authors should explain in the text what the figure shows. Second, the boxplots of each condition should be further apart because there is overlap of individual datapoints between conditions at 0.50. The mean should be observable in the graph since it is what you report in the text. It might be out of ignorance but, what is the black dot inside a grey circle representing?

ANSWER: WE HAVE SEPARATED THE BOXPLOTS AND REDUCED THE SIZE OF THE DOTS SO THAT THERE IS NO OVERLAP OF INDIVIDUAL DATAPOINTS. WE DO NOT REFER TO THE MEAN IN THE TEXT FOR THIS SECTION, SO WE HAVE DECIDED TO KEEP THE MIDDLE LINE OF OUR BOXPLOTS AS THE MEDIAN. WE HAVE ALSO ADDED INFORMATION IN THE FIGURE DESCRIPTION THAT WILL HELP UNDERSTAND THE BLACK DOT WITH A GREY CIRCLE. SEE LINES 362.

- p. 17: report the complete statistics.

ANSWER: WE HAVE REPORTED THE ESTIMATE ± STANDARD ERROR FOR EACH COMPARISON ADDITIONALLY TO THE Z-RATIO AND THE P-VALUES. LINES 377-384.

- Figure 4: The y-axis of graph c) is different than the others. I would eliminate the asterisks from the graphs but I leave it to the authors.

ANSWER: We are keeping the figure as is because otherwise the figure will become confusing, but we appreciate the attention to detail.

- p.19, line 356: report the statistics. Maybe the mean latency and standard deviation could be combined in a Table or graphically with each condition and species. The standard deviation of cats for Control A made me wonder whether you consider all latencies or eliminated outliers. I was also surprised that cats’ latencies were almost twice those of dogs, do you have any idea why?

ANSWER: As it was suggested we have combined the latency information for the three species into a table. As we HAVE OBSERVED here and in our previous experiments dogs are more impulsive and faster than cats. Cats in many cases take their time to make their decisions and many of them not run to the food, like most of the dogs. SEE TABLE 2 IN LINE 403.

- p. 19, line 359: Start with the control conditions as you did in the rest of the results. You should clearly state that you combined the two control conditions.

ANSWER: Done. LINE 421.

Discussion
- I recommend starting the discussion by remembering the reader of your goal and a summary of what you did.

ANSWER: Done. LINES 434-445.

- p. 20, line 377: which ratio? Are you talking about control conditions or test? Be more specific.

ANSWER: Done. LINE 438.

- In line with the difference you observed between male and female dogs, I was wondering if the restriction could have affected cats and dogs differently because of the difference in their weights. All animals were deprived from food the same amount of time regardless their weight. Also, did you control or have any information regarding the animals’ eating routines? That is, did they usually had food always available or did their owners usually give them food at a particular time of the day and a particular amount per day? I wonder if that may affect how animals responded in the task and their motivation.

ANSWER: we did not control or consider the dogs feeding routine, we just asked the owners to deprive them for at least four hours before the test. Since all the dogs finished all trials in one session without any motivational issue, we judged that as a sign that they were hungry enough to perform the task. However, we controlled the weight of the dog and not include individuals to the test below 10 kg, because they might satiate with the food much faster. WE PROVIDE FURTHER EXPLANATION IN THE TEXT (LINE 137-139).

- It would be nice to integrate preference results with latencies – are they in accordance?

ANSWER: WE DECIDED TO REPORT PREFERENCE AND LATENCIES SEPARATELY, AS THEY EACH REQUIERE THEIR OWN EXPLANATION OF RESULTS; HOWEVER, WE HAVE ADDED DETAIL TO THE LATENCIES WHICH WERE A BIT UNDEREXPLAINED AND PROVIDE TWO NEW TABLES (TABLE 2 & 3).

- p. 22, line 439: eliminate “hollow circle”.

ANSWER: these lines were removed.

- P. 23, lines 439 to 442: Is it possible that the donut shape creates an illusion regarding amount?

ANSWER: It is possible that the donut shape with the white circle in the middle and with the black edge creates a visual illusion and gives the false perception to be less than a black circle in the middle with the white edge (circle shape). We added this possibility to the text. See lines 511-517.

- p. 23, line 442 to 446: I felt it was a big jump. Either eliminate this information or make sure you explain why this is important and how is it related to the results.

ANSWER: WE have eliminated the sentence.

- Did you find any effect of presentation order? Is it possible that the animals learned that they would eat the same regardless of what option they chose and, thus, at latter trials start to be indifferent?

ANSWER: WE NOW INCLUDE PRESENTATION ORDER AS ONE OF THE CONFOUNDING VARIABLES TESTED IN THE MODEL (LINE 315 AND REPORT NO EFFECT (LINE 430).

- I suggest a greater focus and discussion on the differences between the animals and humans’ task: 1) the animals were presented with 3D stimuli and the humans with 2D; 2) animals were reinforced for choosing and the stimuli were primary reinforcers whereas the humans were not. 3) Humans were instructed to choose the larger amount thus the task was not analogue. It would be interesting to make humans complete the task without instructions (as the animals).

ANSWER: We also considered these as limitations of this study which are mentioned in the text (line 536-537) and we also included the comment on the stimuli. We also THANK FOR the suggestion about testing humans without instruction. This combined with the use of shapes based on food or other more natural contexts might result an interesting testing option for humans in the future.

I hope you find these comments useful.

**Reviewer #2:** The current study aims at disentangling whether three different species (cats, dogs, and humans) have a preference towards discriminating stimuli based on total amount of said stimuli (by responding to changes differences in a continuous variable; in this case, surface area) or rather on numerosity (by comparing them on the basis of the number of discrete units of the relevant stimuli). In order to do this, the authors employ a paradigm through which the subjects have first to discriminate between two stimuli with the same shape but a different surface area (first with a difference in total area with a ratio of 0.5 and later with a ratio of 0.67), but later on are presented with two stimuli with the same surface area but presented in a different array —most critical to the purposes of this study, in the form of 1, 4, or 8 discrete units. The stimuli presented come in the form of food for the non-human animals and in the form of different figures in a computer screen for humans. Results show that dogs did not show a preference for the larger surface area in the control sessions, humans did, and cats did only do so with the largest different in ratio (0.5). When they compared stimuli of the same surface area, humans seemed to have a bias against a donut-shaped stimulus and dogs did prefer a single stimulus over an array of 8 discrete units, but cats showed no apparent preference for or against any of the arrays of stimuli used.

I believe the current study tackles one of the major confounds when it comes to the study of quantity discrimination, as many of the existing studies in the literature do often equate the discrimination between "numbers" (i.e., amount of discrete units of a given stimulus) to the discrimination between continuous variables. However, I do believe this study does have some issues —both in the methodological and reporting departments— that should be addressed.

What I do believe to be the most important issue in the current study (and the authors briefly touch on it, but not to the degree they ought, in my opinion) is the fact that the existing literature does seem to provide enough evidence to consider that the discrimination between quantities of food or neutral stimuli often provide starkly different results. The most glaring example that comes to mind is Macpherson & Roberts (2013) (doi: 10.1016/j.lmot.2013.04.002), where dogs failed to discriminate between different quantities of food (other than the most simple 1 piece of food vs. 0 pieces), but later on a single dog performed a pilot in which they were able to distinguish ratios of up to 0.75. This is also found (albeit to a lesser degree) in Range et al. (2014) (doi: 10.3389/fpsyg.2014.01299), where dogs were only able to discriminate pieces of food up to a ratio of 0.5. To contrast this one, Rivas-Blanco et al. (number 36 in your citations) showed that they were able to discriminate up to a ratio of 0.8 when it came to dots on a computer screen. So, at the very least for dogs, this difference in paradigm used (quantity discrimination between pieces of food or other neutral stimuli) can potentially impact the outcome of the experiment drastically.

ANSWER: Of course, we agree with the reviewer that dogs’ performance is much better when they are trained. However, some previous studies found that even in a spontaneous two-way food choice task the dogs can discriminate up to 0.6-0.67 ratios (Miletto Petrazzini and Wynne 2016; Ward and Smuts 2007). In the Macpherson and Roberts (2013) study, in the first experiment the food is never visible for the dogs, thus it is not just a spontaneous choice task for them, it also involves their working memory. In the second experiment they trained only one dog for their task. In our study we build in two types of controls – one with ratio 0.5 and one with ratio 0.67 – to ensure that the dogs are able to discriminate between these two amounts of food. Indeed, at the end we found that dogs do not choose above chance level either amount of food, however, we do not consider it as the lack of ability rather than a motivational or impulsivity control problem of the species. Since in the experimental conditions the dogs preferred some of the shapes over the others it's suggest that they do make discrimination and decisions, but it is depend on something else and not on the AMOUNT of food.

Miletto Petrazzini ME, Wynne CDL (2016) What counts for dogs (Canis lupus familiaris) in a quantity discrimination task? Behav Process 122:90-97. https://doi.org/10.1016/j.beproc.2015.11.013

Ward C, Smuts BB (2007) Quantity-based judgments in the domestic dog (Canis lupus familiaris). Anim Cogn 10:71-80. https://doi.org/10.1007/s10071-006-0042-7

The authors do indeed mention differences between "spontaneous" and "trained" quantity discrimination abilities in the introduction, but rather, what seems to me to be the greatest difference between the both is something they do mention later on in the discussion: whenever the subjects are required to differentiate between two quantities of food they are always rewarded, but this is not the case if they are asked to differentiate between two neutral stimuli. As a result, considering the fact that subjects in the study may not be particularly hungry (the authors did ask the owners of cats and dogs to not feed their animals for 4 hours prior to the study, but in my experience, at least dogs are usually fed twice a day, which would mean the animals may have not been food-motivated enough), figuring out the method to obtain the higher amount of food may have not been a priority for the animals, and therefore, the non-human subjects may have not been motivated enough to choose the larger amount of stimuli, regardless of whether or not they had the capability to make this discrimination (this seems to be particularly the case in dogs, but it may have also affected cats' performance to a certain degree as well).

ANSWER: All dogs happily accepted the offered ham and then finished all the trials in one session. This suggested to us that they were well motivated to perform the trials. Of course, we cannot exclude the possibility that the lack of motivation to choose the larger amount of food was behind the lack of significant findings in the control trials. We now include this details in lines 183-185.

As such, I believe the authors should consider the task they presented to the humans and to the non-human animals as two different experiments, and both the discussion and the statistical analyses should be separate. This last point is particularly important, since humans did not only do a different task with different affordances, but also received explicit text-based instructions that the animals (of course) lacked. Therefore, there are three degrees of separation between humans and non-human animals: 1) their species, 2) the task used, 3) the information provided; which I believe should make statistical analyses comparing all three populations inadequate. On this topic, the authors do mention the possibility of testing humans with food or money instead of shapes, but another possibility I reckon worth considering is going the opposite route and using the computer program for all three species (for example, through the use of touch screens). By presenting all three species with the same computer program and having them learn the contingencies of the task through training, it would be possible to equate the participants' information on the task (as well as providing a method to test the non-human animals in this paradigm that does not require the use of language).

ANSWER: thank you for the suggestion. Unfortunately, we are not in a position to apply touch screen monitors and train animals to use them, as we mention that we use animals in people’s households. We acknowledge the advantage of animal training and testing them with neutral stimuli. ADDITIONALLY, we also prefer to test animals in their ‘naturalistic’ environment with ecological relevant stimuli which would be not possible if we want to train them to perform a task like this. Especially cats would be problematic.

Another comment related to statistics, I found it strange that there was no comparison between the performance with each shape in the control conditions. It could be the case that the subjects had different performances based on the shape used, which may or may not be linked to their preferences in the test phase. This is especially relevant in the case of non-human animals, as them struggling (to a higher or lesser degree) to discriminate between the different areas could be partly due to them having issues with one of the shapes in particular.

ANSWER: We had originally considered adding this, but since it was not significant, we removed it for brevity. We have added it again in lines 337 - 340 for the cats, 346 for the dogs and 353-355 for the humans.

Other than this, I found there were some important absences in the way the methodology is reported that preclude (or at least, greatly difficult) a possible replication of the described paradigms. Most notoriously, I believe it would be very important to provide the information the human participants received through the program, as this may have greatly influenced their perception of the task at hand.

ANSWER: We agree. As also R1 mentioned this, we have added the instructions for the human participants exactly as they received them in a supplementary material (See reference in line 273)

Another feature of the experiment that I believe requires further reporting is the arrays of stimuli used in the test phase. It is my understanding that the "circle", "dots", and "crown" shapes are meant to represent different numbers of stimuli (1, 4, and 8, respectively), but I reckon this should be explained clearly in the text. On that topic, I do not understand the inclusion of the "donut" shape, as this would technically represent the same number of discrete stimuli (1) as the "circle" configuration. Not to say it serves no purpose, but the reasoning behind its inclusion should be clearly explained in the text.

ANSWER: This is a good observation by the reviewer. The donut shape purpose was to test another shape which contained only one element and like the circle as much as possible. We specify this now in lines 192-194.

On a minor note concerning the reporting and replicability of the study: the apparent specificity of the areas used for the stimuli (i.e., the fact that the authors did not opt for "round numbers") leads me to believe there may be a practical reason for choosing these areas in particular (e.g., the radius of the circles do happen to be a round number, or this does align with the size constrains of the plates used or the computer screen). If this is the case, this too should be reported.

ANSWER: We added the values of the diameters in lines 196-198, which were the values we originally chose to base the ratio conversion for the rest of the stimuli. We started with a rounded diameter of 4, as the largest area for the cats, calculated the remaining stimuli for these species, and multiplied for the dogs by 2.5.

Additionally, I believe the authors would benefit from making a more throughout review of the existing literature on the topic. Some absences I found worth mentioning are the aforementioned Macpherson & Roberts (2013) paper, and Agrillo et al. (2012) (doi: 10.1371/journal.pone.0031923), which discusses that although humans do make quantity discrimination assessments perfectly (or close to), their response time does slow down as the ratio between the quantities presented gets smaller (which I believe goes along the lines of the reported differences in response time in humans between the control and test phases; as the difficulty of the task increases, they do require more time to make their choice).

ANSWER: thank you for your suggestion, we included the abovementioned paper to the text (line 520).

Finally, one minor style comment, but the authors should consider the way they report numbers. Some sentences do include numbers both as text and as numerals (with a glaring example being line 141-144). On that topic, to my knowledge, when used with abbreviations, numbers are always presented as numerals (e.g., line 130: it should be 10 kg, not ten kg).

ANSWER: We have amended this THROUGHT THE Ms.

Line comments:
Line 56: Consider starting a new paragraph.

ANSWER: Done.

Lines 73-74: I believe "(animals later on)" could be safely removed.

ANSWER: We modified the text as per reviewer’s one comment. Line 65.

Line 76: I would suggest a semicolon after "surface area", for clarity's sake.

ANSWER: Done.

Line 83 & ff.: Consider re-structuring the introduction. The "last resort" hypothesis is mentioned in the paragraph starting with line 63, then there is a paragraph in between, and then this idea is explored again. Additionally, the way I see it, the main topic of the paragraph is that some animals employ discrete numbers while others seemingly do not. Therefore, I would consider re-structuring the paragraph so that it starts exploring that idea.

ANSWER: thank you for the suggestion we’ve rearranged the order of the paragraphs, as visible in line 84.

Line 87: Rivas-Blanco et al. 2020 (citation number 36 in your paper) could arguably also be included when you mention dogs here. Dogs were provided with numerical data, but the controls suggest that they made their comparisons based on continuous information.

ANSWER: thank you for the suggestion, the abovementioned paper now is included earlier in the text (line 101).

Line 93: The change is topic is rather drastic, consider providing a segue.

ANSWER: We think it's fine the way it is now.

Line 97-98: I feel the topic of Weber's law should be introduced earlier.

ANSWER: We have removed the mention of Weber’s law since it would be more complex theory that we make of use of and instead replace it with the phenomenon we explore in this paper, as can be seen in lines 98-99.

Line 102 & ff.: Same as before, I should consider restructuring the introduction so that the ideas discussed flow naturally into each other. The introduction is rather short, so there should be no need of a paragraph starting with "as mentioned above" or any variation thereof by carefully paying attention to the structure.

ANSWER: We removed as “mentioned above.”

Line 119: A brief overview of the conditions used and the reasoning behind them before the methods would prove very useful for the reader.

ANSWER: Good point. We added a few lines providing the reasoning behind the conditions in the last paragraph of the introduction (Lines 121-124)

Line 130: Please provide reasoning for the weight requirements for the dogs. Also "in the case of dogs, be over ten kg" > "in the case of dogs, they had to be over 10 kg".

ANSWER: Done. See lines 137-139.

Line 131: The proper abbreviation for "hour" is "h".

ANSWER: Done.

Line 136: "testing sessions" rather than "occasions of testing"?

ANSWER: Done. Line 150.

Line 141 & ff.: It would be really useful if the authors could provide a subject list with some basic information for the subjects (age, sex, breed in the case of dogs...).

ANSWER: We have amended the text to provide the details for the cat and dog samples with individual identifiers (file named “subject_data.csv”) IN AN ONLINE REPOSITORY (see lines 151-152).

Line 153: "a) they should" > "they should a)".

ANSWER: We removed “they should” alltogether

Line 166: While I agree on the notion, this seems like an odd way to start the paragraph.

ANSWER: We modified this text by moving the sentence further inside the paragraph (lines 180-181).

Lines 167-168: I do not think I understand the authors' reasoning. If the authors really concur with the notion that the type of food used for testing has no effect on cats' performance, then why would it be necessary to check their preferences.

ANSWER: THE REVIEWER IS CORRECT; the sentence was contradictory. We have amended the text to justify why we tested different kinds of foods (lines 176-179).

Lines 177 & ff.: I reckon more details should be provided in this paragraph.

ANSWER: We have added more detail to this PARAGRAPH (lines 173-175).

Line 182: Maybe "quantity discrimination abilities" rather than "ability of quantity discrimination"?

ANSWER: Done. Line 187.

Line 191: Consider starting a new sentence instead of using a semicolon.

ANSWER: Done.

Lines 200-201: However, the dogs were not provided with chairs to account for this. Care to elaborate?

ANSWER: We now specify why cats needed a chair, while dogs did not (lines 220-222).

Lines 218-220: This should be moved to the cats and dogs' section. It is the same for all species, but it would prove more useful over there (since it comes earlier in the paper).

ANSWER: We reduced the text but have kept it separate by species as there are some minor differences.

Lines 221-222: Consider mentioning that these controls are meant to be similar to the ones used for cats and dogs. I reckon it makes it easier for the reader.

ANSWER: We have amended this sentence. Line 282.

Line 227: I reckon it would be easier if the paragraph started by emphasizing the fact that this was done exclusively in humans (e.g., rather than "for the the human participants", something like "as opposed to dogs and cats", or something to that effect. Additionally, it would be interesting to know the reasoning behind this additional control.

ANSWER: We removed references to “In humans” etc, as now the section is under a “Human” subheading; also, we have modified the text regarding the additional control for clarity (lines 274-275).

Line 240: The 4-hours food deprivation was brought elsewhere. I do not have a particular preference about the location of that snippet of information, but I reckon it should be kept in only one place.

ANSWER: We have removed the second instance.

Line 245: May be worthwhile to further explain the "gently hold it there from behind"; not sure if it is completely clear.

ANSWER: Done. Line 240.

Line 272: Consider rewording, this makes it sound like the dogs were also on a chair.

ANSWER: We reworded this. Lines 243-244.

Line 288: Maybe "all analysis were" rather than "all analyses was"

ANSWER: Done. Line 290.

Line 288 & ff.: On the models below you mention having checked for the potential influence of random effects, but here you add together data from all participants, which could lead to pseudo-replication issues. Why?

ANSWER: We mention that R2 did not change upon the addition of random effects, so we combined the participants data. We specify this in lines 308-310.

Line 296 & ff.: It would be useful to provide the formula of the resulting model.

ANSWER: We provide the formula. Line 308.

Line 301: Remove one of the two periods.

ANSWER: Done.

Line 304-306: Not sure if I understand what this means. Care to elaborate?

ANSWER: We’ve reworded the sentence to make it clearer. Lines 314-317.

Line 307-309: Same as above, this does not account for differences within each individual.

ANSWER: We opted for a different analytical approach regarding latency, but we expanded our description in lines 318-325.

Line 330-331: Consider rephrasing: the program failed to record, not the glitch.

ANSWER: Done. Lines 357-358.

Lines 362-363: Please specify what is meant by "comparing differently sized circles".

ANSWER: We rephrased the sentence. Lines 408-409.

Line 383-383: As mentioned above, please specify what is meant with "circles of different areas".

ANSWER: Done. Lines 443-444.

Line 387: Consider removing "their test".

ANSWER: Done.

Line 406 & ff.: Only one of the combinations was significant. That makes it more likely that we may be facing a spurious correlation. I think this should be brought up in the text.

ANSWER: We have corrected for multiple comparisons, and remained highly significant, so we don’t agree that this is a spurious result.

Lines 424-426: Would not an equal reliance on both factors imply that they would prefer a higher number of discrete forms in the screen? That is to say, given that the participants were (as far as I understand) instructed to pick the largest option, and considering that in the test session both stimuli had the same surface area, the only distinguishing factor remaining would be number. Or put in other words: if there are no differences in surface area (and thus discriminating on the basis of continuous variables was not possible), the only other relevant variable would be number.

ANSWER: As the text mentions in line 271, participants were asked to choose “larger black area”, so no confusion should have happened regarding the task. Additionally, what we tested was whether they chose the largest individual area.

Line 436: I think it would be better to start a new sentence instead of using a semicolon.

ANSWER: Done.

Line 439-446: I am not sure how much value this provides to the paper.

ANSWER: We changed the text to make it more relevant for the current context. See lines 507-516.

Line 447: Comma after "humans".

ANSWER: Done.

Lines 458-460: As I mentioned in the beginning, given that this is the case, I reckon it is not justified to put all species in the same model.

ANSWER: While humans and companion animals were tested in different settings, it is often the case that you need to make some methodological compromises in order to be able to perform such comparisons. We think we have acknowledged this appropriately in our limitations in lines 530-535. Any points the reviewer would like to add, we can consider.

Line 469 & ff.: I would try to tone down the claims. I am not sure whether this could be said with the results of the current study alone.

ANSWER: We toned down our claims. See lines 539-541.
