# Supplementary material for: Continuous or discrete magnitudes? A comparative study between cats, dogs and humans
Source: PLoS One. 2025 Oct 1;20(10):e0331924. doi: 10.1371/journal.pone.0331924 (PMC12488003; doi:10.1371/journal.pone.0331924)
Supplement: Attachment — Submitted filename: Response to Reviewers_R2.docx [file pone.0331924.s005.docx]

**Reviewers' comments to the Author**

**Reviewer #1:** Great job improving the clarity of the manuscript! Overall, the authors did a careful revision and adequately responded to my previous comments. The procedure is much clearer. However, I still have some comments and suggestions but much minor compared to the previous ones. As a general comment, the authors tend to use sentences and paragraphs that are too big and with too many ideas (this is even more salient in the discussion).

***R:*** *We have tried to divide the introduction and discussion into paragraphs so that each of them discusses only a single idea.*

Abstract
p-2, line 31-32: be more specific than “some preference” or, at least, specify a preference for what.

***R:*** *We have added that the preference was for certain shapes, particularly the circle. See line 32.*

p.2, line 34: at this point, the reader does not know what is the control condition. Instead of using the name of the condition, describe it as you did in the rest of the sentence.

***R:*** *We have added a description of the condition. See line 35.*

Introduction
p. 4, line 68: processing numerical information involves a larger cognitive load compared to what? Also, what “this strategy” is referring to? You did not describe any strategy in the previous sentence.

***R:*** *We have added information and amended the sentence. See lines 69 and 70.*

p.4, lines 73 to 76: Divide the sentence in key ideas. As it is, it is difficult to understand the message.

***R:*** *The sentence has been rewritten to be shorter and clearer. See lines 75-77 and 77-79.*

p.4, line 82: why do you considered the use of number rather than of the continuous magnitudes a bias?

***R:*** *Thanks for noticing. We’ve reworded this sentence in line 85.*

p.5, line 91: species rely on the ratio between what? the numerosity? the surface area? of the two stimuli. The information inside brackets is important to understand the results so it should be a regular sentence.

***R:*** *We have modified the sentence as suggested in lines 93-96.*

p.5, line 97: decompose the sentence, there are too many ideas and information. How did the animals choose (response) and what was the correct response? Choosing the three item-set? End the sentence at this point. Then you can start the next sentence with results. This is only a suggestion:” Cats reliably learned to choose (...). But, when surface area was controlled, rats were indifferent between the two stimuli suggesting their previous preference was based on the surface are and not on quantity.”

***R:*** *Done, we have separated and clarified this section.*

p.5, line 100: consider substituting “above 0.5 ratios” for “when the ratio between the two stimuli was greater than 0.5”. In the same sentence, I did not understood what the authors meant with “both numerical and continuous magnitude differences”.

***R:*** *We have substituted the sentence as suggested and we have added information for clarification. Other changes have been done as per the above comment, but this specific one can be found in lines 105-110.*

p.5, line 105: Explain what you mean by “counterbalanced” in this case.

***R:*** *Counterbalanced was not the correct word replaced for the correct procedure description in lines 111-114.*

p.6, line 114: The clarity of this paragraph can be increased.

***R:*** *We have summarized the paragraph to improve its clarity. See lines 115-129.*

Methods
p.6, line 127 to 129: weight at least 10 kg instead of “over 10kg” and the information that comes next should not be part of the same paragraph. Suggestion: “This last constraint serve to….”.

***R:*** *Thanks for noticing this rather important detail. We have corrected the section as suggested in line 138.*

p. 7, line 141: “details of”

***R:*** *Correct. We have amended the sentence which is in now line 152.*

Table 1: I believe there is a better way to present the table. Since there is much more information regarding cats and dogs, I would suggest a table for those and the information regarding the humans can either be in a separate, simpler table or in the text. Below there is a table suggestion with only some information just to exemplify. You may need to switch the lines with columns due to the space occupied when more info is added.
Age Sex
M (SD) Range Female Male
Neutered Intact Neutered Intact
Cats 6.4 (3.7) 1 – 14 9 1 12 4
Dogs 4.1 (3.1) 1 – 13 11 4 9 4

***R:*** *Thank you for the suggestion, we have removed humans from the table and added their information to the text. The table is now presented in a similar manner to the reviewer’s example. See new Table 1 after line 155.*

p. 10, line 189: the ratio between the surface areas of the two food portions was 0.5

***R:*** *We have changed the sentence as suggested. See line 204*.

p.14, line 249: the experimenter asked the participant’s initials, age….

***R:*** *Done. Line 264.*

p. 14, line 260: what is S1?; “completed trials of three different conditions”

***R:*** *We have modified the sentence. S1 is the supplementary information file which contains the instructions for the human participants and can be found at the end of the document, under Supporting information. Although the link to the actual file may not have been correctly provided by the submission system. In any case, we made sure the S1 is in the new revision.*

p.14, line 263: “a total of 28 trials, x of each condition”

***R:*** *We have now added the number of trials for each condition. Lines 278-280.*

p.15: Four trials of Control A plus 4 of Control B plus 6 of Experimental do not comprise 28 trials as stated before. I anticipate there were two trial-blocks?!

***R:*** *We have removed this as it was from a previous version of the MS and did not make sense anymore.*

p.15, line 272: Suggestion: “If participants took more than 10 s to click on either option (i.e., the maximum choice latency allowed), the trial was cancelled and the next trial started immediately.

***R:*** *We have used the reviewer’s suggestion to modify some parts of our original sentence in lines 288-292.*

Results
Instead of “latency of choice”, use “latency to choose”. Be careful when using “female dogs’ choice for the larger” or “human participants’ choice of the larger”, it adds complexity to the information.

***R:*** *We have changed “latency of choice” to “latency to choose” as suggested. We have also restructured the sentences mentioned to make them less complex (for instance, in line 340).*

Discussion
In general, paragraphs are too long and with too many ideas. Try to divide them.

***R:*** *We have divided the paragraphs throughout the discussion to make them more concise.*

p.23, line 426: substitute extremely by significantly

***R:*** *We have substituted the word in line 451.*

p.23, line 428: According to the first part of the sentence, I was expecting the second part to be something like: while cats did not showed a preference for any particular shape or while cats were indifferent between shapes.

***R:*** *We have modified the sentence according to the reviewer’s expectation in line 453-454.*

P23, line 430: “in the control condition” can be eliminated because you already said it in the beginning of the sentence.

***R:*** *We have removed this part. Line 456.*

p.25, line 463: did you meant within each sample?.

***R:*** *The reviewer is right, we have changed it to within in line 501.*

p.25, line 466: where the ratio between…

***R:*** *Done. Lines 505-506.*

p.25, line 466: The difference in preference observed between male and female dogs…

***R:*** *Thank you for the suggestion, we have changed the sentence. Lines 506-507.*

p.25, line 467: Did you check if there was any effect similar to that reported by Simona, Maria?

***R:*** *We added some text and a caveat regarding the small sample size after the contrast of our study to that of Simona, Maria’s. See lines 511-513.*

p.25, line 473: dividing by sex; it might be possible; I think the conclusion of the sentence is odd. I would rather say that any differences may not be possible to observe in your sample because of its small size, mainly when dividing by sex.

***R:*** *We agree that the former sentence was odd. We have integrated the reviewer’s suggestion with the original idea to make it more understandable. See lines 516-520.*

p.25, line 476: the discrimination is not between ratios is between two stimuli with a certain ratio between them. Be careful with this among the entire manuscript.

***R:*** *We have modified the sentence to make this point clear (line 522), and changed other instances along the manuscript where this may have been pointed out.*

p.25, line 479: please explain what is the result suggests each of the two possibilities. What led you to those possibilities? It seems you are jumping a step in the rationale.

***R:*** *We have made more explicit the results that led us to this possibility but also have amended the text to focus only on the main one by removing lines 529-532.*

p.26, line 490: …and their ratio change which….

***R:*** *Done. Line 540.*

p.26, lines 496 and 497: three repetitions of the word making.

***R:*** *We have now changed two of these repetitions to make the sentence more clear. For instance, in line 547.*

p.26, line 498: I believe words are missing from the sentence.

***R:*** *The reviewer is correct. We have added a few words to complete the sentence. See lines 548-549.*

p.27, line 504: latencies

***R:*** *Done. Line 555.*

p.27, line 506 and 507: when the control trials involved circles of different areas

***R:*** *We have changed the wording as suggested. Lines 558.*

**Reviewer #2:** General comments
The main issue of this manuscript (and one that remains unchanged in this version) is that the authors do not exercise enough caution when comparing the different species. The paradigm used for both dogs and cats is similar enough to allow for a direct comparison, but humans get a completely different paradigm that involves no food, as well as instructions (that the animals, of course, do not get). Therefore, I think statistical analyses including data from all three species are inapropriate, and comparisons between humans and non-human animals should be kept to a minimum.

***R:*** *We agree with the reviewer. As suggested, we have opted for simpler statistical analyses for each individual species, avoiding direct comparisons between humans and non-human animals. Please see the new analysis in lines 313-332 and other changes throughout the manuscript.*

The end of the introduction still feels rather bare, I think a paragraph in which the authors discuss the paradigm they used and how this connects to what the experiment is meant to test would be an important addition to the text.

***R:*** *As requested by R#1, we greatly revamped the next to last sentence to make it clearer. This now includes a statement of what the article is meant to evaluate. Please see lines 115-129.*

Finally, the authors talk about ecological relevance being the driver behind the methods described. However, dogs are scavengers in nature, and they're well-suited for periods of scarcity. Under the abundance of food experienced by most house pets, optimal foraging (or, in this context, choosing the larger quantity) would arguably rarely be a priority. Particularly if, as the authors mention in the introduction, numerosity happens to be a particularly cognitively loading skill. These considerations do not need to be added to the manuscript, but I still thought they were worth discussing.

***R:*** *Given the concerns brought on by the reviewer, we opted to remove the statement about the food being an ecologically relevant stimulus and present it as can be read in lines 126-128.*

In-line comments
103-107: Consider restructuring this sentence, it is somewhat hard to parse.

***R:*** *We have broken up the sentence into multiple ones and restructured it, we hope this improves the clarity.*

105: "When" should be in lowercase.

***R:*** *We have corrected this mistake. Line 111.*

115: "and an experimental one".

***R:*** *We have changed this as suggested. Line 125.*

126-129: Please consider rephrasing, it remains rather hard to follow.

***R:*** *We have reworded the section so that it is less dense and can be followed easier. See lines 123-129.*

185-186: I reckon it would be easier to follow if you used some sort of index to designate the three different sizes (e.g., "small", "medium", and "large"). The same designation could be carried later on when the comparisons are described.

***R:*** *We have incorporated this designation into the text so that the description of the methodology is easier to follow. See lines 198-200.*

210-211: I reckon it would read better as "something dogs did not need due to their height", or something to that effect.

***R:*** *We appreciate the suggestion, we have used it to change the sentence. Lines 225-226.*

233: As I mentioned last time, to my knowledge, numbers are always used as numerals when the unit is used as an abbreviation. So it can be "2 meters", "two meters", and "2 m", but not "two m".

***R:*** *The reviewer is right; we have corrected the sentence to “2 m”. See line 248.*

235-237: I understand it is important to keep the subjects from getting satiated in order to increase the number of potential trials within a session, but would this not affect the animal's perception of the choice they made in the control conditions? Even assuming every individual would go for the highest amount of food under optimal conditions, if they were allowed to eat the same amount of food regardless of whether they choose the larger amount or not, then why would any of the choices be any better than the other? I reckon this deserves further comment, both in this section and the discussion.

***R:*** *We have added a limitation regarding the potential biases introduced by allowing the animals to consume the chosen stimuli. See lines 583-586.*

262: It is unclear what sort of counterbalance is being discussed here.

***R:*** *We have described this in more detail to improve clarity.*

273: "jump" > "skip"

***R:*** *Done. Line 292.*

298: Not sure if I understand how the "shape" variable was added to the model. The way I understood the sentence, the trial type would be a factor with 6 levels (one for each of the potential combination of stimuli), and the shape would be each of these stimuli. But this would be a problem when building the model matrix since, for instance, no data point could simultaneously have "circle vs. donut" in the "combination variable" and "dots" in the shape variable. So I suppose I did not quite get what you did here. Care to elaborate?

***R:*** *The reviewer is correct; we have analyzed our model carefully and realized it poses problems due to some of its interactions. We have explored multiple alternative ideas and have decided to use a simpler form of analysis, binomial tests, for the results of the experimental trials; these give us equivalent results to the model while reducing complexity. This also removes the issue caused by comparing between the three species. See lines 313-332.*

348: Not sure what is meant by the "(0.0001)"

***R:*** *This number referred to the proportion of errors in relation to the total number of trials. However, we realize it could be confused for a p-value and have thus removed it in line 370.*

458: Dogs in the wild live in fission-fusion societies, so I do not think the designation of "pack-living animal" is deserved. Either way, please provide references of such claims.

***R:*** *While wild dog societies do have fission-fusion dynamics, we have seen that most studies investigating these species and their social structure describe their groups as “packs”. We have added references to this section in line 493.*

458-459: You may want to read this paper: https://doi.org/10.1007/s00265-017-2339-8 . It goes in the same direction to what you mention about "protecting one big piece of food from other conspecifics".

***R:*** *We thank the reviewer for the suggestion we have read and incorporated the paper into the discussion as it offers evidence of food monopolization in dogs in lines 488-499.*

467: Is "Simona, Maria (43)" a citation? Not sure if this is clear in the text.

***R:*** *Yes, this is a citation, but we had not formatted it properly. It has been fixed now, as can be seen in lines 508 and 513.*

471-472: This sentence does not connect well with the rest of the paragraph, and I reckon it should be deleted.

***R:*** *We have rephrased this paragraph greatly, and in the processes, removed the sentence the reviewer makes reference to, in lines 516-520.*

474: A new paragraph should start in "seemingly".

***R:*** *We have separated this section into a new paragraph, line 521.*

496: I reckon "tricky" could be safely removed.

***R:*** *We have removed the word as suggested. Line 547.*

516: Which results in particular?

***R:*** *We have specified which shape preferences lead us to believe that the convex hull does not have a significant effect in choice in lines 569-573.*
