# Supplementary material for: Continuous or discrete magnitudes? A comparative study between cats, dogs and humans
Source: PLoS One. 2025 Oct 1;20(10):e0331924. doi: 10.1371/journal.pone.0331924 (PMC12488003; doi:10.1371/journal.pone.0331924)
Supplement: Attachment — Submitted filename: Response to Reviewers_R3.docx [file pone.0331924.s006.docx]

**Reviewer #1**: Most comments you will find in this review are minor and mostly related to wording.

Introduction
p.3, line 61: The definition of continuous magnitudes should be available earlier since it was already used a considerable number of times.

*R: The reviewer is correct. We have introduced a short definition in the first mention of continuous magnitudes, in line 47-48.*

p. 4, line 94: consider specifying “…the ratio between the smaller and the larger stimuli”. If the ratio is between the larger over the smaller, the larger the ratio, the easier it is to discriminate.

*R: We have replaced the first instance of ‘ratio’ with ‘difference’ as the sentence may be confusing. A larger ratio between the stimuli (100/99 = 0.99) is more difficult to discriminate than a smaller one (100/80 = 0.80). See line 99.*

p. 4, line 103: They could be based their choice on both cues and when one of them is changed (in this case, surface area) it is as if a completely new stimuli are presented and animals are indifferent.

*R: We have added this argument into the description of the study, since we agree it is a possible explanation for the behavior shown. See lines 109-110.*

p.5, line 112: Suggestion: “the use of numerosity with the use of continuous magnitudes of stimuli when discriminating between quantities and, the ones that do, entail methodological differences….”.

*R: We have replaced the text with the reviewer’s suggestion. See lines 91-94.*

Methods
p. 8, line 158: Substitute “S” by “SD”.

*R: Done.*
p.9, line 185: Eliminate “one, four, or eight, …” from the sentence and add “ : ” following the parenthesis.

*R: Done.*
p. 9, line 191: Edit the sentence to make clear the use of the letters in line 192: “…three different diameters (d) … different surface areas (a)…”

*R: Done.*
p.13, line240 – 241: Confirm if you don’t need to add some commas.

*R: No commas, but we slightly rephrase the sentence.*

p.13, line 246: “… and thus were given a 10-minute break.”

*R: Done.*
p. 13, line 254: Eliminate the word “study”.

*R: Done.*
p.14, line 272: “12 for the experimental condition.”

*R: Done.*
p.15, line 275: Suggestion: “In Control A, the surface areas were 29.22 cm2 and 14.61 cm2 thus, the ratio between the two stimuli was equal to 0.5.”

*R: Done.*

Analysis
p. 16, line 307: I think the authors meant to use “e.g.,” instead of “i.e.,”.

*R: Done.*

Results
p. 19, line 362: Edit the sentence: “… none of the shapes was chosen significantly more than the others, …”

*R: Done.*
p. 19, line 364: “… an effect on choice with subjects showing a significant preference for the circle over the crown…”

*R: Done.*
p. 19, line 367: “… revealing a significant preference for the circle …”

*R: Done.*
p. 20, Table 2: In the title, I believe the authors meant “Control and Experimental conditions” instead of trials.
*R: Done.*

Discussion
p. 23, line 426: I am not sure but I believe it is more accurate to use “faster” rather than “quicker”.

*R: Done.*

p. 24, lines 438 – 440: I was wondering if the fact that cats are generalist predators should not make them more sensitive to differences in shape and thus, I would expect the exact opposite result. The argument might be used both ways. To maximize their hunting they should be capable of clearly distinguishing between different preys.
*R: In these sentences, our claim is not that they are not capable of distinguishing between shapes, but rather that cats do not do so in the present experiment.* *If every cat prefers a different shape, this may mask this ability. It is possible that each individual had its own preferred shape, but the number of experiments conducted under Experimental Conditions is insufficient to test this reliably. We know mention this more clearly in the text. See lines 459-463.*

p. 24, line 444: You don’t need the sentence starting with “We can…”. Start immediately with: “One possible explanation is that….”.
*R: Done.*

p.24, line 446: “a) they were well fed and b) they could…, food would be always available at the end of all trials”

*R: Done.*
p. 24, line 448: “Another possible explanation”

*R: Done.*
p. 24, p461: If the same phenomena was observed in cats, how can you use it as a possibility for a result found in dogs but not in cats?!

*R: We have reviewed the cited reference again and decided to remove its mention from the text since it is more focused on monopolization according to food quality, instead of size or shape like we tested in our protocol, and we understand it is confusing for the readers. See lines 487-490.*

p.24, line 473: Was the difference between neutered and intact dogs significant? If not, it is not a difference. Moreover, I think you did not have a sample size big enough to say this.

*R: We agree, the difference was not significant due to our limited sample size. Thus, we have decided to rewrite this sentence to clarify the findings. See lines 503-506.*

p.24: In future studies, you can also use stimulus different from food with the animals.

*R: We have added this to the limitations in lines 569-570.*

**Reviewer #2**: I believe the authors have done a great job re-vamping the stats and addressing most of the issues that were found before. However, I still have a few minor comments remaining.

Line 69: "processing" is already used in this sentence, please consider a synonym.

*R: Done.*

Line 75: Please consider rephrasing the "They found that continuous [...]". I reckon "These studies found that continuous [...]" would be clearer and easier to follow.
*R: Done.*

Lines 112-114: This sentence should go before the new paragraphs in which the authors talk about previous studies with cats and dogs since, the way the sentence is formulated, it seems to imply that examples of such studies will be discussed straight after that. It would also serve to illustrate the different approaches that are used and why it would be necessary to use the same method for comparative purposes.
*R: We have moved this sentence further up to highlight the differences in previous studies, and we have also restructured the penultimate paragraph of our introduction so that the ideas are easier to follow in lines 91-94.*

Line 114: On that topic, I am not sure if you want "they have relied on testing only a few species" to be a part of your message. Cats and dogs have been tested already. The one thing you provide over previous studies is a protocol that controls for shape of the stimulus and the comparative aspect of testing two species within the same protocol. Then, the question could also be why those two species in particular, etc.
*R: We have rewritten this section to emphasize the things mentioned by the reviewer (the comparison between species and the controlling of shape) instead of the focus on which species have been tested in lines 91-94.*

Line 238: "Cueing" was correct. "Cueing" is "providing cues". "Queueing" is "standing in line".
*R: Done.*

Line 272: "for experimental condition" → "for the experimental condition"
*R: Done.*

Lines 282-283: Consider rewording, not sure if I follow.
*R: Done.*

Lines 452-457: Please pay more attention to the literature: both "wild" and "free-ranging" dogs are dogs (and as far as I understand, they refer to the similar populations in this case), while dholes are not. Therefore, the reference about dholes seems wholly out of place, and so any wider references to "canids". Please consider reading further on studies done on free-ranging dogs.
*R: We have removed references to other canids and focused solely on studies that discuss free-ranging dogs and their social behavior. See lines 478-482.*

Line 461: You do state that "similar phenomena was also observed in cats", yet in this study, cats did choose at random whenever presented with different shapes, which would go against the explanation you laid out in the lines above. Please elaborate on this.
*R: We have reviewed the cited reference again and decided to remove its mention from the text since it is more focused on monopolization according to food quality, instead of size or shape like we tested in our protocol, and we understand it is confusing for the readers. See lines 486-490.*

Lines 461-463: Did the authors on those study also conclude that the largest item would facilitate monopolizing that resource? Please elaborate.
*R: The authors of the referenced work describe another test in which a much smaller amount of food but with more pieces does not induce monopolization. However, it is important to consider this test was conducted only in dyads and not in a group setting. We now address this and well as the need for more studies. See lines 486-490.*

Line 469: You do continue with the same idea on the next paragraph. It may not be the best place to end the paragraph.
*R: Done.*

Lines 485-488: That being the case, would you not expect humans to show indifference for the different stimuli in much the same manner as cats did? Please elaborate.

*R: We believe our use of “idiosyncratically guided” was an oversimplification of the processes at hand and we have modified the sentence to clarify why we think the human participants had such preferences. See lines 519-521.*
